# Comparative Evaluation of the Effects of Amorphous Silica Nanoparticles on the Erythrocytes of Wistar Normotensive and Spontaneously Hypertensive Rats

**DOI:** 10.3390/ijms24043784

**Published:** 2023-02-14

**Authors:** Zannatul Ferdous, Ozaz Elzaki, Sumaya Beegam, Nur Elena Zaaba, Saeed Tariq, Ernest Adeghate, Abderrahim Nemmar

**Affiliations:** 1 Department of Physiology, College of Medicine and Health Sciences, United Arab Emirates University, Al Ain P.O. Box 17666, United Arab Emirates; 2 Department of Anatomy, College of Medicine and Health Sciences, United Arab Emirates University, Al Ain P.O. Box 17666, United Arab Emirates; 3 Zayed Center for Health Sciences, United Arab Emirates University, Al Ain P.O. Box 17666, United Arab Emirates

**Keywords:** silica nanoparticles, hypertension, erythrocytes

## Abstract

Silica nanoparticles (SiNPs) are one of the most widely used nanomaterials. SiNPs can encounter erythrocytes and hypertension is strongly linked to abnormalities in the functional and structural characteristics of erythrocytes. As little is known about the combinatorial effect of SiNP-hypertension interactions on erythrocytes, the aim of this work was to study the effects triggered by hypertension on SiNPs induced hemolysis and the pathophysiological mechanism underlying it. We compared the interaction of amorphous 50 nm SiNPs at various concentrations (0.2, 1, 5 and 25 µg/mL) with erythrocytes of normotensive (NT) and hypertensive (HT) rats in vitro. Following incubation of the erythrocytes, SiNPs induced significant and dose-dependent increase in hemolysis. Transmission electron microscopy revealed erythrocyte deformity in addition to SiNPs taken up by erythrocytes. The erythrocyte susceptibility to lipid peroxidation was significantly increased. The concentration of reduced glutathione, and activities of superoxide dismutase, and catalase were significantly increased. SiNPs significantly increased intracellular Ca^2+^. Likewise, the concentration of the cellular protein annexin V and calpain activity was enhanced by SiNPs. Concerningly, all the tested parameters were significantly enhanced in erythrocytes from HT rats compared to NT rats. Our results collectively demonstrate that hypertension can potentially exacerbate the in vitro effect induced by SiNPs.

## 1. Introduction

Advancement in nanotechnology served as a pioneer for advanced science in the field of biology and medicine [1]. Nanomaterials, in recent years, have gained huge popularity due to their unique physicochemical properties such as small size, large surface area to volume ratio, high reactivity, high carrier capacity, and easy variation of surface properties [1]. Engineered nanoparticles (NPs) are being increasingly used for various biomedical purposes such as drug delivery, antimicrobial agents, molecular imaging, and biomedical sensing [1,2].

Silica nanoparticles (SiNPs) are among the most frequently encountered NPs in our daily life as they are used as additives in cosmetics, drugs, and food [3]. The easily modifiable chemistry in silica for optimal biocompatibility and biodistribution has promoted its extensive application in bio imaging, gene therapy and drug delivery [3]. Moreover, SiNP-based drugs are favored over traditional drugs due to their increasing availability [4]. Due to the extensive application, human exposure to SiNPs can occur via inhalation, ingestion, topical, or direct systemic injections. Furthermore, these NPs can be distributed in blood and interact with circulatory cells. Hence, the benefit of SiNPs in biomedical applications is tempered by serious concerns about their potentially undesirable effects, particularly on blood compatibility and stability [5,6].

We have previously demonstrated that the incubation of amorphous SiNPs at various concentrations (1–125 µg/mL) with mouse erythrocytes in vitro caused dose-dependent hemolysis, in addition to altered oxidative stress markers, apoptosis, and increased cytosolic Ca^2+^ [7]. Similar in vitro analysis evaluating the effects of amorphous SiNPs on human erythrocytes, concluded that energy metabolism disorder in addition to oxidative damage, contributed to the hemolytic effects of SiNPs [8]. Mechanisms of hemolysis were also reported based on the functional group, for instance, the electrostatic interaction of the Si-OH group with membrane proteins or the quaternary ammonium of phosphatidylcholine present in the cell membrane resulting in membrane destabilization [9]. Studies based on hemolysis assays by other commercialized NPs such as silver NPs (AgNPs), titanium dioxide (TiO_2_), cerium oxide, and polymer NPs, also demonstrated size-dependent adsorption, uptake, and dose-dependent hemolytic activity in erythrocytes in vitro and/or in vivo, in addition to cardiovascular and systemic toxicity [8,10,11,12]. Wang et al. found that red blood cells (RBC) and hemoglobin concentrations increased in rats treated with graphene quantum dots [13]. Furthermore, we have previously shown that intraperitoneal administration of SiNPs causes proinflammatory and procoagulant responses in vivo and in vitro [14]. In agreement with this, Corbalan et al. demonstrated that in vitro exposure of human platelets to amorphous SiNPs, induced oxidative effects leading to platelet aggregation [15]. Although a few studies have examined the hemolytic effect of SiNPs, there are much fewer investigations on impeding effect of SiNPs on erythrocytes of populations of cardiovascular pathologies such as hypertension.

Hypertension is a very common healthcare problem in adult and elderly populations. The erythrocytes in hypertensive patients face multiple risks such as oxidative stress, endothelial dysfunctions, and a decrease in erythrocyte plasma membrane fluidity, which lead to mechanical and rheologic damage of erythrocytes, thus sustaining a high blood pressure [16]. Hence, understanding what happens when nanomaterials interact with blood components is a critical step when evaluating health risks for hypertensive patients. Limited experimental evidence from cardiopulmonary-compromised animal models has shown increased mortality from chronic exposure to simulated particle/gaseous urban air pollution in spontaneously hypertensive rats [17]. A few studies have demonstrated that pulmonary exposure to engineered NPs is capable of aggravating cardiovascular dysfunction via mechanisms including systemic inflammation, coronary artery dysfunction, metabolic derangement, autonomic dysregulation, and oxidative stress [18,19,20]. Additionally, some studies reported abnormalities in the coagulation and fibrinolytic pathways, as well as in platelets and the endothelium, among hypertensive experimental models and patients [21,22,23]. However, the influence of hypertension on SiNP-induced pathophysiological effects on erythrocytes remains poorly understood.

Based on the above-discussed reports, we hypothesize that a hemolytic effect can be influenced by chronic cardiovascular disorders such as hypertension. Consequently, the aim of this study was to assess and compare the effect of SiNPs on erythrocytes of normotensive (NT) and hypertensive (HT) rats at various concentrations (0.2, 1, 5, and 25 µg/mL) on hemolysis, uptake of SiNPs by erythrocytes, oxidative stress, intracellular calcium, annexin V, caspase 3, and calpain.

## 2. Results

### 2.1. Erythrocyte Analysis by TEM

**Figure 1:**
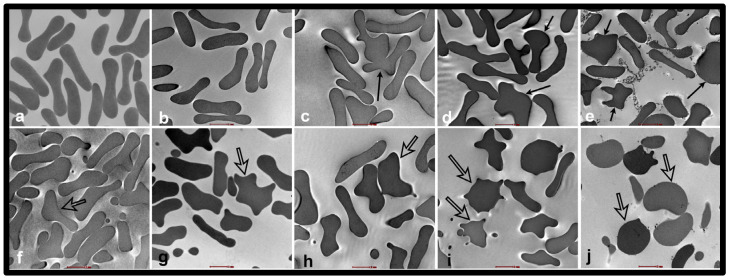
Transmission electron micrographs of the erythrocytes (RBCs) of normotensive (NT) ((**a**–**e**): (**a**) NT-saline; (**b**) NT 0.2; (**c**) NT 1:0; (**d**) NT 5.0; (**e**) NT 25 µg/mL nanoparticles) and hypertensive (HT) ((**f**–**j**): (**f**) HT-saline; (**g**) HT 0.2; (**h**) HT 1:0; (**i**) HT 5.0; (**j**) HT 25 µg/mL nanoparticles) rats after treatment with silica nanoparticles (SiNPs). Abnormal RBCs (thin arrows) were observed in NT rats after the addition of nanoparticles at a concentration of 1, 5 or 25 µg/mL. In contrast, the RBCs of HT rats show abnormal shapes (empty arrows) even when treated with saline (**f**). The addition of nanoparticles increased the number of deformed RBCs. Scale bar = 2 µm.

**Figure 2:**
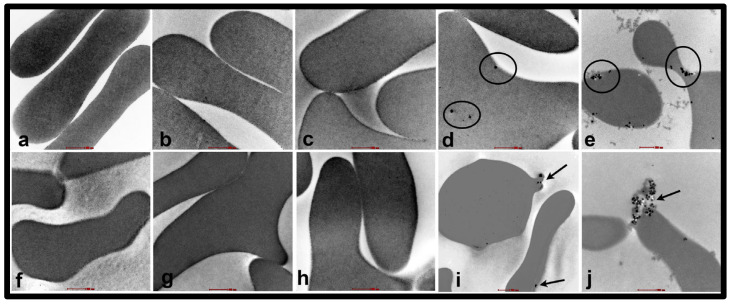
Transmission electron micrographs of the erythrocytes of normotensive (NT) ((**a**–**e**): (**a**) NT-saline; (**b**) NT 0.2; (**c**) NT 1:0; (**d**) NT 5.0; (**e**) NT 25 µg/mL nanoparticles) and hypertensive ((**f**–**j**): (**f**) HT-saline; (**g**) HT 0.2; (**h**) HT 1:0; (**i**) HT 5.0; (**j**) HT 25 µg/mL nanoparticles) rats after treatment with silica nanoparticles (SiNPs). A strong interaction between RBCs and nanoparticles were observed after treatment with nanoparticles at a concentration of either 5 or 25 µg/mL. The 50 nm SiNPs entered RBCs and caused destruction and eventual sloughing (arrow) of red blood corpuscles in HT rats (**i**,**j**). The extent of RBC damage is less severe after attachment of nanoparticles (empty circles) onto RBCs of NT rats. Scale bar = 2 µm.

**Figure 3:**
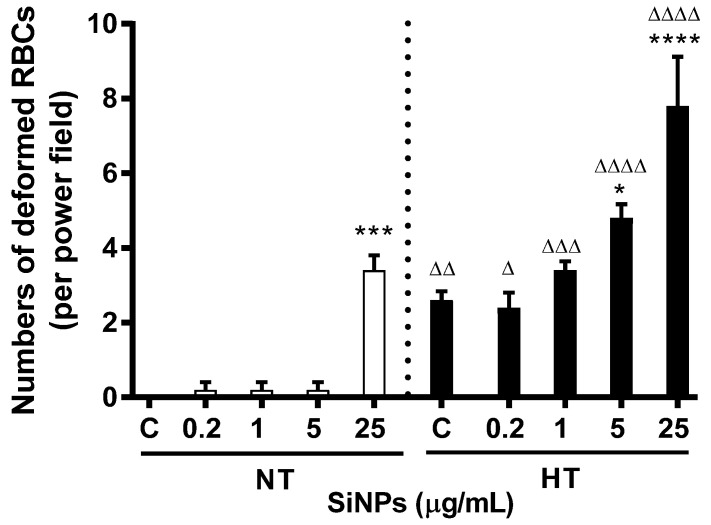
Numbers of deformed red blood cells (RBCs) (per power field, ×11,100) of normotensive (NT) and hypertensive HT) rats after treatment with various concentrations (µg/mL) of silica nanoparticles (SiNPs). Data are mean ± SEM (*n* = 5 in each group). Statistical analysis by one-way analysis of variance (ANOVA) followed by the Holm-Sidak multiple comparisons test. * *p* < 0.05, *** *p* < 0.001, **** *p* < 0.0001 vs. respective control and ^Δ^ *p* < 0.05, ^ΔΔ^ *p* < 0.01, ^ΔΔΔ^ *p* < 0.001, ^ΔΔΔΔ^ *p* < 0.0001 vs. respective concentration in NT group.

TEM images of erythrocytes incubated with SiNPs at concentrations of 0.2, 1, 5, and 25 µg/mL are shown in Figure 1 and Figure 2. The erythrocytes of NT rats treated with 1, 5, and 25 µg/mL of SiNPs showed abnormal shapes (Figure 1 and Figure 2b–e). The degree of abnormality increases with the concentration of the NPs, with the erythrocytes of NT rats treated with 25 µg/mL nanoparticles having the highest number of deformed RBCs (Figure 3). In contrast, the erythrocytes of HT rats showed abnormal structure after treatment with saline alone, a feature not seen in the erythrocytes of NT rats. The development of abnormal structure by the erythrocytes of HT rats was further enhanced after the addition of SiNPs (Figure 1 and Figure 2g–j). The damage induced by NPs includes tissue sloughing (Figure 2j). It is noteworthy that HT rats’ erythrocytes treated with a concentration of either 5 or 25 µg/mL were grossly deformed (Figure 2i,j and Figure 3).

### 2.2. Effect of SiNPs on Erythrocyte Hemolysis

**Figure 4:**
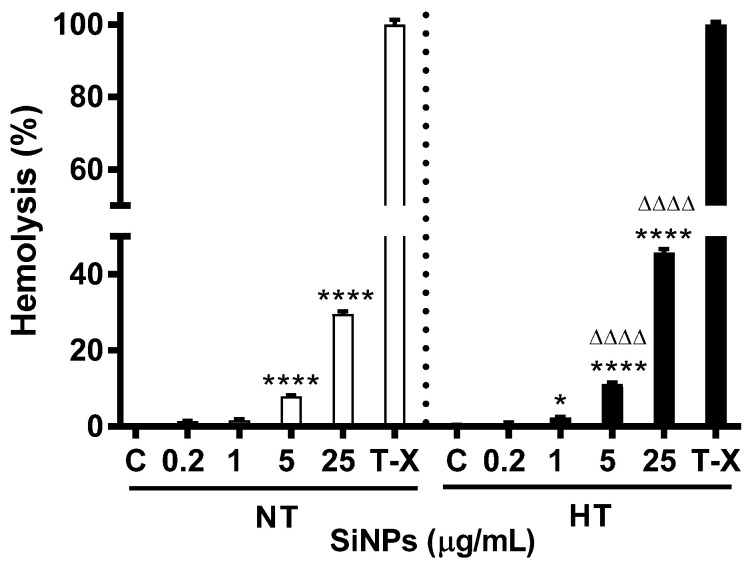
Hemolytic effect of various concentrations (µg/mL) of silica nanoparticles (SiNPs) in incubated normotensive (NT) and hypertensive (HT) rat erythrocytes. The results are expressed as % of positive control (0.1% Triton-X 100). Data are mean ± SEM (*n* = 8 in each group). Statistical analysis by one-way analysis of variance (ANOVA) followed by the Holm–Sidak multiple comparisons test. * *p* < 0.05, **** *p* < 0.0001 vs. respective control and ^ΔΔΔΔ^ *p* < 0.0001 vs. respective concentration in NT group.

Figure 4 shows the hemolytic effect of SiNPs on erythrocytes. Incubation of erythrocytes with SiNPs caused dose-dependent hemolysis. Compared with their respective controls the effects were significant at concentrations of 5 µg/mL (*p* < 0.0001) and 25 µg/mL (*p* < 0.0001) for the NT group and at a concentration of 1 (*p* < 0.05), 5 (*p* < 0.0001) and 25 µg/mL (*p* < 0.0001) for the HT group. The hemolytic effect was also significant at doses 5 (*p* < 0.0001) and 25 µg/mL (*p* < 0.001) for HT animals compared with the respective dose in NT animals.

### 2.3. Effect of SiNPs on LPO and GSH Concentrations and on SOD and CAT Activities

**Figure 5:**
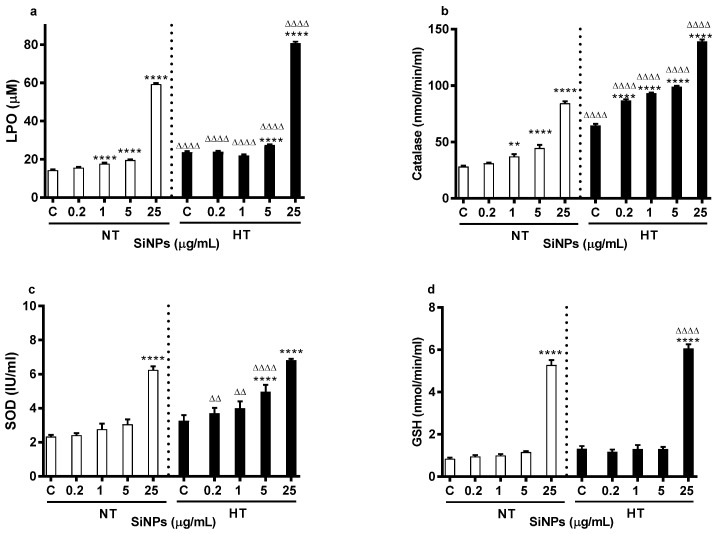
Effect of various concentrations (µg/mL) of silica nanoparticles (SiNPs) on lipid peroxidation concentration (LPO, (**a**)), catalase activity (**b**), superoxide dismutase activity (SOD, (**c**)) and reduced glutathione concentration (GSH, (**d**)) measured in the incubation medium of erythrocytes obtained from normotensive (NT) and hypertensive (HT) rats. Data are mean± SEM (*n* = 8 in each group). Statistical analysis by one-way analysis of variance (ANOVA) followed by the Holm-Sidak multiple comparisons test. ** *p* < 0.01, **** *p* < 0.0001 vs. respective control and ^ΔΔ^ *p* < 0.01, ^ΔΔΔΔ^ *p* < 0.0001 vs. respective concentration in NT group.

The concentrations of LPO and GSH and activities of SOD and CAT are shown in Figure 5. LPO was used to assess the susceptibility of erythrocytes’ lipid peroxidation in vitro. Compared with their respective controls, the LPO concentration was dose-dependently increased by the incubation of erythrocytes with various concentrations of SiNPs for both the NT and HT groups (Figure 5a). While the significance for the NT group was achieved at 1, 5, and 25 µg/mL (*p* < 0.0001), it was attained at concentrations of 5 and 25 µg/mL (*p* < 0.0001) for erythrocytes from HT animals. However, compared with respective doses in the NT group, there was a significant increase in the HT group at all tested concentrations (*p* < 0.0001).

Likewise, compared with their respective controls, there was dose-dependent increase in catalase activity for both NT and HT groups (Figure 5b). The level of significance was achieved at 1 (*p* < 0.01), 5 (*p* < 0.0001), and 25 µg/mL (*p* < 0.0001) for the NT group and at 0.2, 1, 5, 25 µg/mL (*p* < 0.0001) for HT group. Compared with the respective dose in the NT group, the catalase activity was significantly increased for HT groups at all tested concentrations (*p* < 0.0001).

Figure 5c shows dose-dependent increase in the activity of SOD. However, significance was achieved only with the highest concentration in the NT group, and at 5 and 25 µg/mL (*p* < 0.0001) for the HT group, compared with their respective control. Compared with respective doses in the NT group, the SOD activity was significantly increased at 0.2 (*p* < 0.01), 1 (*p* < 0.01), and 5 (*p* < 0.0001) µg/mL for the HT group.

Figure 5d shows the effect of SiNPs on the concentration of GSH. Compared with the respective control and respective dose in the NT group, the concentration of GSH was significantly increased for the HT group at the highest concentration i.e., 25 µg/mL (*p* < 0.0001).

### 2.4. Effect of SiNPs on Lactate Dehydrogenase (LDH) Activity

**Figure 6:**
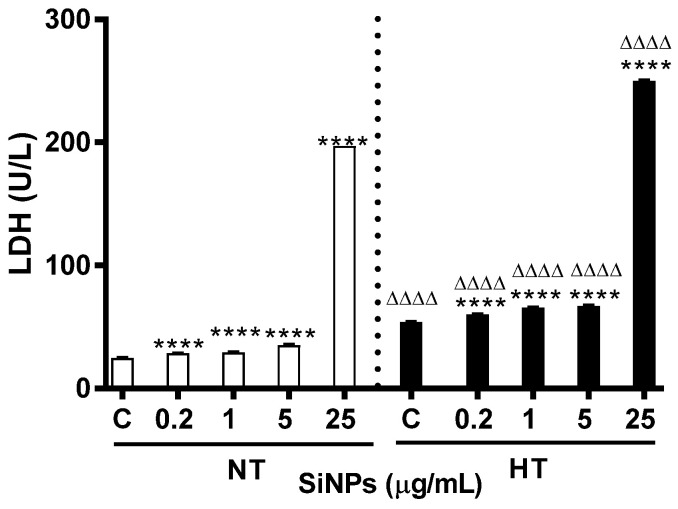
Effect of various concentrations (µg/mL) of silica nanoparticles (SiNPs) on lactate dehydrogenase concentration in incubated normotensive (NT) and hypertensive (HT) rat erythrocytes. Data are mean± SEM (*n* = 6 in each group). Statistical analysis by one-way analysis of variance (ANOVA) followed by the Holm–Sidak multiple comparisons test. **** *p* < 0.0001 vs. respective control and ^ΔΔΔΔ^ *p* < 0.0001 vs. respective concentration in NT group.

Figure 6 shows the effect of various concentrations of SiNPs on the LDH activity of incubated erythrocytes in the NT and HT groups. A significant dose-dependent increase was observed among both groups at all tested concentrations (*p* < 0.0001), compared with their respective control. Interestingly, significance was also seen in the HT group when compared to respective doses in the NT group for all tested doses (*p* < 0.0001).

### 2.5. Effect of SiNPs on ATPase

**Figure 7:**
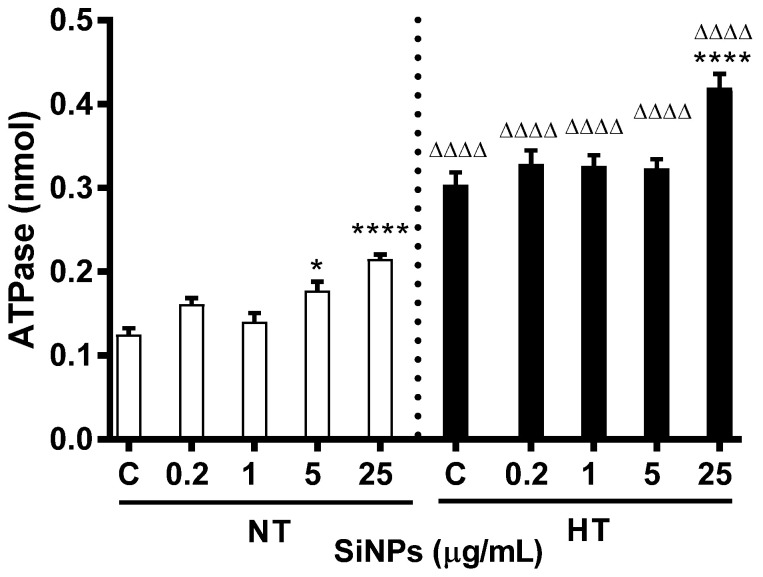
Effect of various concentrations (µg/mL) of silica nanoparticles (SiNPs) on ATPase activity measured in incubated erythrocytes of normotensive (NT) and hypertensive (HT) rats. Data are mean± SEM (*n* = 8 in each group). Statistical analysis by one-way analysis of variance (ANOVA) followed by the Holm–Sidak multiple comparisons test. * *p* < 0.05, **** *p* < 0.0001 vs. respective control and ^ΔΔΔΔ^ *p* < 0.0001 vs. respective concentration in NT group.

Concentrations of ATPase in incubated erythrocytes are shown in Figure 7. While a significant increase was seen at 25 µg/mL (*p* < 0.0001) for the HT group, the level of significance was observed at 5 (*p* < 0.05) and 25 µg/mL (*p* < 0.0001) for NT group. However, compared to the respective doses in the NT group, the HT group showed significance at all tested concentrations (*p* < 0.0001).

### 2.6. Effects of SiNPs on Intracellular Calcium

**Figure 8:**
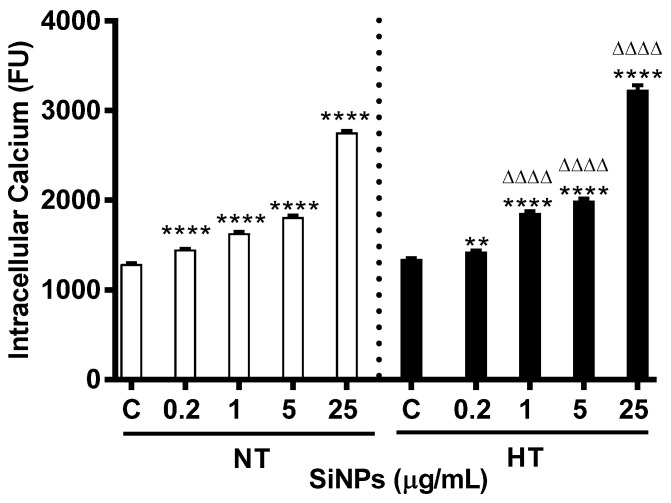
Effect of various concentrations (µg/mL) of silica nanoparticles (SiNPs) on intracellular calcium concentration measured in incubated erythrocytes of normotensive (NT) and hypertensive (HT) rats. Data are mean± SEM (*n* = 8 in each group). Statistical analysis by one-way analysis of variance (ANOVA) followed by the Holm–Sidak multiple comparisons test. ** *p* < 0.01, **** *p* < 0.0001 vs. respective control and ^ΔΔΔΔ^ *p* < 0.0001 vs. respective concentration in NT group.

Figure 8 illustrates the effect of various concentrations of SiNPs on cytosolic calcium concentration from Fluro3 fluorescence. The incubation of erythrocytes with SiNPs caused a significant dose-dependent increase in cytosolic calcium concentration compared with respective control for both groups at all tested concentrations (*p* < 0.0001). However, compared to respective doses in the NT group, intracellular calcium was significantly increased at doses of 1, 5, and 25 µg/mL (*p* < 0.0001–*p* < 0.01) for the HT group.

### 2.7. Effect of SiNPs on Annexin V-Binding

**Figure 9:**
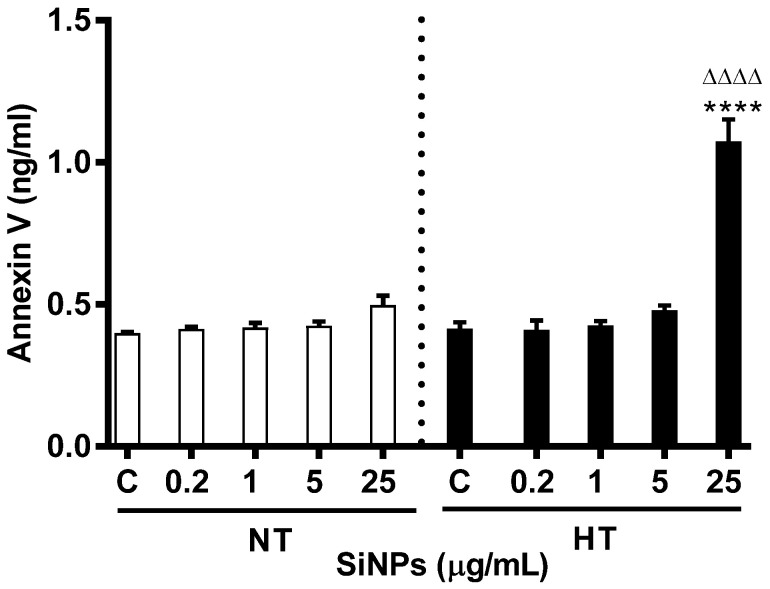
Effect of various concentrations (µg/mL) of silica nanoparticles (SiNPs) on the concentration of bound Annexin V in the incubation medium of erythrocytes of normotensive (NT) and hypertensive (HT) rats. Data are mean± SEM (*n* = 8 in each group). Statistical analysis by one-way analysis of variance (ANOVA) followed by the Holm–Sidak multiple comparisons test. **** *p* < 0.0001 vs. respective control and ^ΔΔΔΔ^ *p* < 0.0001 vs. respective concentration in NT group.

Exposure of phosphatidylserine at the cell surface was estimated from bound annexin V. Incubation of erythrocyte with SiNPs triggered annexin V binding, as shown in Figure 9. Overall, there was no effect of SiNPs on Annexin V binding in incubated erythrocytes except at the highest concentration i.e., 25 µg/mL (*p* < 0.0001) for the HT group, compared to the respective control and respective dose in the NT group.

### 2.8. Effect of SiNPs on Calpain Activity

**Figure 10:**
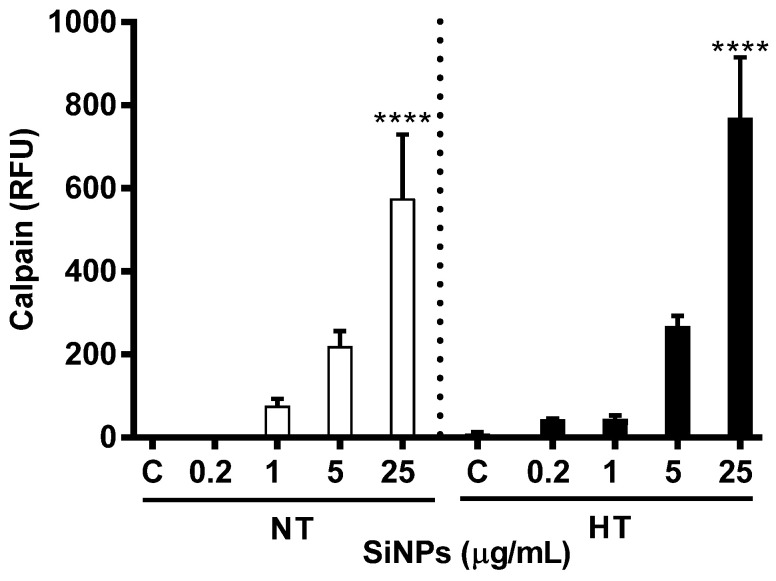
Effect of various concentrations (µg/mL) of silica nanoparticles (SiNPs) on calpain activity measured in incubated erythrocytes of normotensive (NT) and hypertensive (HT) rats. Data are mean± SEM (*n* = 6 in each group). Statistical analysis by one-way analysis of variance (ANOVA) followed by the Holm–Sidak multiple comparisons test. **** *p* < 0.0001 vs. respective control.

The assessments of activated calpain in cytosol upon incubation with erythrocytes with various concentration of SiNPs is represented in Figure 10. Compared with the respective control, a significant increase in calpain activity was only achieved at the highest concentration (25 µg/mL) for both the NT and HT groups (*p* < 0.0001).

## 3. Discussion

Underlying cardiovascular complications are increasingly prevalent and may represent susceptible subpopulations that need to be considered within toxicity assessments of emerging exposures to NPs. In this work, we assessed and compared the in vitro effects of amorphous SiNPs on NT and HT Wistar rat erythrocytes and showed that SiNP-induced hemolysis, oxidative stress, increase cytosolic Ca^2+^, annexin V binding, and calpain activity are aggravated by hypertension.

With the development of nanotechnology, drug delivery systems based on nanomaterials exhibit great potential applications in the fields of diagnosis and therapy [24,25]. The wide range of applications makes it possible for them to enter the body by various routes including oral, inhalation, intravenous, hypodermic, or intramuscular administration [26]. Consequently, NPs can be translocated and distributed in the bloodstream and affect vascular homeostasis [3,27]. Hence, it is essential to understand the interaction of NPs with erythrocytes. More importantly, the scarcity of studies in this field makes it even more essential to investigate the influence of common cardiovascular diseases like hypertension on the latter interactions. Alterations in blood rheological variables during hypertension have been extensively reported [23,28,29]. Our present in vitro comparative erythrocyte study is relevant to previous in vivo studies which demonstrated that exposure to SiNPs is associated with cardiovascular toxicity in addition to impaired vascular homeostasis, inflammation, endothelial dysfunction, and prothrombotic state [18,30]. We incubated the rat erythrocytes with saline or SiNPs from the same source; selecting the size (50 nm), an incubation time point (4 h), and concentrations (0.2, 1, 2, and 25 µg/mL) similar to previous in vitro studies [31,32]. The rationale of various concentrations (0.2, 1, 5, and 25 µg/mL), selected in the present study, to human exposure, were explained in our previous in vitro studies evaluating nanotoxicity and blood compatibility of mice erythrocytes incubated with SiNPs [7]. In fact, we used a concentration (0.2 µg/mL) much lower than the lowest concentration used (1 µg/mL) and excluded the highest concentration (125 µg/mL) of the previous study [7]. In addition, data from our previous study comparing systemic and vascular responses to 50 nm and 500 nm SiNPs, indicated smaller-sized NP caused more pronounced effect than the larger NPs. Hence, we choose smaller-sized SiNPs (50 nm) in the present study.

Hemolysis is proven to be the expression of NPs induced cytotoxicity. In this regard, various coating AgNPs had a hemolytic effect on mice and human blood, and TiO_2_ had a direct hemolytic effect in rabbit erythrocytes [10,33,34]. Our previous studies on the interaction of mice erythrocytes with amorphous SiNPs (50 nm), at a dose of 25 µg/mL also demonstrated a significant dose-dependent hemolysis [7]. The hemolytic activity of SiNPs exposed erythrocytes has been demonstrated to be dependent on the presence of negatively charged silanol groups [35]. However, what remains unclear is whether preexisting cardiovascular complications such as hypertension can influence this hemolytic effect. Therefore, erythrocytes from the hypertensive model were selected to fully understand the hemocompatibility of amorphous SiNPs. Our hemolytic assay results determined that erythrocytes of HT rats exhibited an exacerbated dose-dependent hemolytic response compared to erythrocytes from NT rats.

Moreover, TEM analysis has been performed following the incubation of erythrocytes from NT and HT rats at various concentrations of SiNPs to obtain more information about the uptake and localization of SiNPs within erythrocytes. Considerable interaction between erythrocytes of NT and HT rats was observed when the concentration of the NPs was between 5 and 25 µg/mL. In line with this, our previous studies showed that diesel exhaust nanoparticles and SiNPs are taken up by erythrocytes [7,36]. Geiser et al. [37], in studies of TiO2 NPs on lung cells and erythrocytes, showed that particle uptake in cells was by diffusion or adhesive interactions. Though our study does not clearly define the mechanism of entry of SiNPs into the cell, it could be possible that, given their very small size, the SiNPs were able to enter the erythrocytes via diffusion as proposed earlier [38,39]. Interestingly, more severe lesions to the erythrocytes of HT rats compared to NT rats were observed in addition to deformed RBCs. Dead erythrocytes from HT rats were seen after the incubation with the highest concentration of SiNPs. The latter findings indicate a combinatorial damaging effect of NPs and hypertension.

We further assessed and compared the effect of SiNPs on the biomarkers of oxidative stress, including LPO, GSH, SOD, and CAT. Following the occurrence of oxidative stress, erythrocyte membranes are susceptible to LPO that involves cleavage of polyunsaturated fatty acids at their double bonds leading to the formation of aldehydes, malondialdehyde, and 4-hydroxynonenal [40]. Our current data showed a significant dose-dependent increase of LPO in erythrocytes of the HT group compared to the NT group. The latter could be attributed to added rheological stress to erythrocytes due to hypertension in addition to lipid peroxidation derivative substances that have the potential to modify the structural and functional integrity of cells [40]. Oxidative stress results from an imbalance between reactive oxygen species (ROS) and the antioxidant defense system. Erythrocyte antioxidants including SOD, GSH, and CAT are major circulating antioxidants in the oxidative stress defense system that control the biological effect of ROS [41]. SOD catalyzes the conversion of superoxide radicals to hydrogen peroxide while GSH quenches the free radicals by serving as an electron donor and CAT converts hydrogen peroxide to water and oxygen [42]. Our data show that incubation with SiNPs results in a significant dose-dependent increase in SOD activity, GSH concentration, and CAT activity compared to the respective controls. In fact, the CAT and SOD activities, and GSH concentration were potentiated for erythrocytes from HT rats compared to the same dose of NT rats, suggesting that HT generated stress on erythrocytes can further exacerbate the influence of SiNPs. Overall, these results reinforce the relationship between SiNPs interaction and alteration in oxidative stress and antioxidant enzyme activities and highlight the fact that preexisting hypertension can augment the impact of SiNPs.

Cell membrane integrity is usually measured by LDH activity in cell medium, which is an effective indicator for irreversible cell damage. According to our previous study performed in RBCs of healthy mice, the incubation of erythrocytes with SiNP showed a significant dose-dependent increase in LDH activity and at concentrations 25 and 125 µg/mL SiNPs [7]. Interestingly, using RBCs from rats, our present results showed a significant increase even at lower tested concentrations i.e., 0.2, 1, and 5 µg/mL SiNPs for both the experimental group. This could be attributed to the variability of the effect of SiNPs on erythrocytes of different species. Furthermore, the LDH activity was more important in erythrocytes of HT rats compared to NT ones. LDH activity showed a dose-dependent increase both in human endothelial cells and the erythrocyte supernatants after the amorphous SiNP incubation [19]. These results showed evidence that SiNPs exerted hemolytic effects by increasing the erythrocyte membrane permeability and promoting red cell rupture. Our, as well as others’, results, collectively indicate that the exacerbation of LDH activity in erythrocytes exposed to SiNPs is not only particle and dose-dependent, but also depends on the preexisting condition like hypertension.

It is well known ATPases act as a molecular motor that uses the energy of ATP hydrolysis to power reactions such as cellular metabolism, protein assembly and trafficking, replications, transcriptions, and ion pumping [43]. The ATPase assay is a membrane assay that indirectly measures the activity of the efflux transporter which is responsible to mediate the transport of substrates across cell membranes against a concentration gradient. Our current data show that the total ATPase activity is increased at all concentrations in both the NT and HT erythrocytes incubated with SiNPs. Interestingly, while a significant increase was observed only at higher concentrations for the NT erythrocytes, the effect was substantially enhanced in erythrocytes of HT animals compared to their respective concentrations of NT erythrocytes. Endocytosis was considered the common way involved in the interaction of SiNPs with the red cell membrane, and their occurrence relied heavily on localized ATPase regulating erythrocyte membrane deformability [44,45,46]. In adult patients with hypertension and prehypertension lowered ATPase activity was documented in erythrocytes [45]. Similarly, the ATPase pump was significantly inhibited in the samples from hypertensive children [47]. Lizhen et al. also demonstrated decreased ATPase activity in erythrocytes and suggested that amorphous SiNPs could reduce the capacity of energy generation in human erythrocytes [8]. Inconsistently, increased ATPase activity in our present study could be attributed to the type of erythrocyte, and the size of NPs, in addition to the rheological effect of hypertension. In fact, the latter response could be a counter mechanism to combat the altered oxidative stress.

In order to further understand the molecular mechanism and pathway leading to enhanced hemolysis in the HT group of erythrocytes compared to the NT group of erythrocytes incubated with SiNPs, we further assessed intracellular Ca^2+^ concentration, Annexin V binding, and calpain activity. Mature erythrocytes are highly specialized cells that lack normal cell organelles, such as nuclei and mitochondria, which are vital for the regulation of apoptosis, an innate mechanism of programmed cell death [48]. The senescence involved in mature erythrocytes is characterized by distinct changes in shape and plasma membrane with translocation of phosphatidylserine from the inner leaflet of the cell membrane, and is termed an eryptosis [49,50]. The latter has been evaluated using various techniques including cytofluorometric analysis [51]. However, unlike apoptosis of erythroblast that is caspase-dependent, the mature erythrocyte is driven into eryptosis by an increase in intracellular calcium which in turn triggers activation of calpain [50]. Apoptosis-mediated cell death via SiNPs exposure has previously been demonstrated in hepatocytes (HL-7702), where the increasing concentration of NPs resulted in a significant increase in cell death [52]. Our previous study demonstrated increased cytosolic Ca^2+^ and annexin V binding in mice erythrocytes following incubation with SiNPs in addition to a significant stimulation of caspase 3 and aspartate-specific cystine proteinases; factors that promote apoptosis [7]. Typical features of eryptosis including cell shrinkage, membrane blebbing, and phosphatidylserine externalization have been reported in erythrocytes exposed to Ca^2+^ ionophore ionomycin or A23187 [53,54]. Annexin V is a Ca^2+^-dependent cellular protein that has the ability to bind with phosphatidylserine and is used as an apoptotic marker when exposed to the outer leaflet of the cell membrane [53,55]. Additionally, it has been shown that increased calcium activity is due to the activation of Ca^2+^ permeable cation channels which is, in turn, triggered by erythrocyte injury including oxidative stress [49]. Our present study data show a significant and dose-dependent increase of intracellular Ca^2+^ and annexin V binding with augmented responses in erythrocytes from HT animals compared to a similar dose of NT animals. Calpain, a calcium-dependent cystine protease, similar to caspases, exits as proenzymes and is reported to play a role in eryptosis [56]. Our data show that the incubation of NT or HT erythrocytes with SiNPs, results in a significant increase in calpain activity compared with control, and this increase could be attributed to the elevated cytosolic Ca^2+^. Our findings corroborate with a previous study which demonstrated that changes in intracellular calcium led to the activation of calpain [57].

To conclude, the interaction of SiNPs was comprehensively investigated in an in vitro model of NT rat erythrocytes and compared with HT rat erythrocytes. Our data reinforce that SiNPs caused hemolysis all tested concentrations. It is well known that the hemolytic activity and resulting systemic and vascular effect of most NPs is composition, concentration, structure, size, and shape-dependent [58]. Although the methods whereby the NPs interact with erythrocytes to cause hemolysis are still debated, different mechanisms have been proposed, including interactions with the erythrocyte membrane, cellular uptake, and internalization [58]. Nanoparticles cellular uptake and internalization pathways mainly rely on active endocytosis mediated by vesicles [59]. In addition, passive transportation through red blood cell membrane has been demonstrated in recent studies [60,61]. One of the prominent mechanisms potentially applicable to many NPs, including SiNPs, is the direct interaction of NPs with the erythrocyte membrane which can lead to injury, detrimental morphological changes, and cytoskeletal distortions, resulting in erythrocyte aggregation, reduced deformability, and oxidative stress [35,62]. Previous studies suggested that the SiNP-cell interaction, uptake, and toxicity are directly proportional to the number of reactive silanol groups exposed on the NP surface which in turn depends on the size and geometry of the NPs [35,63]. Erythrocytes investigated in our current study demonstrated 50 nm SiNPs induced oxidative stress and increased ATPase activity, cytosolic calcium, annexin V binding, and calpain activity. The small size (providing a large surface-to-volume ratio), in addition to the composition, concentration, and shape of 50 nm spherical SiNP used may contribute to effective NP uptake by erythrocytes with consequent increased interaction and toxicity. Furthermore, our data reveal that pre-existing hypertension may enhance the hemolytic effects, oxidative stress, lipid peroxidation, and eryptosis following SiNPs exposure. This exacerbation of effects warrants more extensive investigation in hypertensive subjects daily exposed to SiNPs. It is also noteworthy to understand how the physicochemical characteristics of nanoparticles, such as size, shape, coating, and functional group, influence biological interactions and functions [64,65]. Hence, more extensive studies are needed using various properties in order to understand the effect and underlying mechanism of SiNPs on erythrocytes.

## 4. Materials and Methods

### 4.1. Particles

Amorphous SiNPs (50 nm) were purchased from Polysciences, Inc. (Warrington, PA, USA). Data regarding their characteristics were recently published [15,66,67]. As described in the manufacturer’s data sheet (Polysciences, Warrington, PA, USA), the commercially available negative surface charged SiNPs used in our present study were amorphous solid nonporous pure silicon dioxide with SiOH (non-functionalized) surface group. These nanoparticles were synthesized by a precipitation process. Using the same type of SiNPs from the same source, we and Corbalan et al. reevaluated and confirmed the size stated by the supplier using a transmission electron microscope [15,66]. In addition, Corbalan et al. found a high negative zeta potential (more negative than −30 mV) of the SiNPs [15,67].

SiNPs were suspended in normal saline (NaCl 0.9%). To minimize aggregation, the stock particle suspensions were always sonicated (Clifton Ultrasonic Bath, Clifton, NJ, USA) for 15 min and vortexed for 30 s before their dilution and incubation with erythrocytes.

### 4.2. Animal Handling and Blood Collection

Male Wistar (9–10 weeks old, weighing about 250 ± 10 g) and spontaneously hypertensive (9–10 weeks old, weighing about 280 ± 10 g) rats were housed in a room at a temperature of 22 ± 2 °C, relative humidity of about 60%, with a 12 h light–dark cycle, with free access to water and standard pellet diet ad libitum. All experimental procedures were in accordance with the protocols of the Research Advisory Committee Review Board of United Arab Emirates University.

Animals were anesthetized intraperitonially with sodium pentobarbital 30 mg/kg, and then blood was drawn from the inferior vena-cava with a syringe prewetted with 4% sodium citrate and collected in EDTA (4%) containing tubes. The blood sample was then processed according to the type of experiment conducted.

### 4.3. Preparation of Erythrocytes

The erythrocytes were prepared using a previously described method [36,68]. Briefly, collected rat blood was mixed by gentle inversion of the tube and centrifuged at 1200× *g* for 15 min. The plasma supernatant was discarded, and the erythrocytes were washed 4 times by suspending them in 0.9% NaCl followed by centrifugation at 1200× *g* for 10 min. The final suspension consisted of 5% by volume of erythrocyte in saline. Flat bottom 12 well plates were used to incubate 900 µL of erythrocyte with 100 µL of SiNPs (0.2, 1, 5, and 25 µg/mL). The plate was positioned in an MS 3 digital microtiter shaker (IKA WER KE GmbH & CO, Staufen, Germany) and rotated at 300 rpm for 30 min at room temperature, under shaded light. After incubation, the samples were transferred to a 1.5 mL Eppendorf tube and were centrifuged at 1200× *g* for 5 min. The resulting supernatant was collected for hemolysis assay, oxidative markers assays, intracellular calcium, Annexin V binding, ATPase activity, and calpain activity. The deposited erythrocytes were fixed with Karnovsky’s fixative (2% paraformaldehyde and 2.5% glutaraldehyde in 0.1 M phosphate buffer at 7.2 pH) for transmission electron microscopy (TEM). The deformed erythrocytes were quantitated and expressed as the number of RBCs per field at a magnification of 11,100.

### 4.4. Hemolysis Assay

The hemolysis assay was performed according to a previously reported technique [7,69]. Briefly, erythrocytes incubated with SiNPs (0.2, 1, 5, 25 µg/mL) were centrifuged as described above. After that, 90 µL of the supernatant was added to a 96-well plate, and the amount of hemoglobin released was determined spectrophotometrically at a wavelength of 540 nm. The percent hemolysis was calculated using the linear equation *y* = m*x* + *c* where %hemolysis (*x*) = ((sample optical density (*y*) − negative control optical density (*c*))/(positive control optical density − negative control optical density) (m)) × 100 [36,68].

Erythrocytes retrieved from NT and HT rats were processed for TEM according to a previously described method [7]. Briefly, the cells were washed with 0.1 M phosphate buffer and for 1 h at room temperature on rotamixer and post-fixed with 1% osmium tetroxide in 100 mM cacodylate buffer. After that, cells were dehydrated in a series of graded ethanol concentrations, infiltrated with Agar 100 epoxy resin, embedded into resin-filled molds, and polymerized at 65 °C for 24 h. Blocks were trimmed and ultrathin sections were obtained by an ultra-microtome (Leica, Mikrosysteme GmbH, Vienna, Austria). Ultrathin sections were collected on 200 mesh copper grids and were contrasted with uranyl acetate and followed by lead citrate double stain. The grids were examined and photographed at variable magnification with a Tecnai Spirit BioTWIN TEM (FEI, Holland) [7,70,71].

### 4.5. Measurements of Levels of Lipid Peroxidation (LPO), Catalase (CAT), Superoxide Dismutase (SOD), and Reduced Glutathione (GSH)

Following incubation of erythrocytes with SiNPs or normal saline (control), the mixture was centrifuged, and the resulting supernatants were subjected to oxidative damage/stress marker assays including LPO, CAT, SOD, and GSH. NADPH-dependent erythrocyte membrane LPO was measured as a thiobarbituric acid reactive substance using malondialdehyde as standard (Sigma-Aldrich Fine Chemicals, St. Louis, MO, USA. GSH concentration (Sigma Chemicals, St. Louis, MO, USA), and CAT activity (Activity kit, Cayman chemicals, Ann Abror, MI, USA) were analyzed spectrophotometrically according to methods described by the manufacturers. SOD activity was measured as the conversion of nitroblue tetrazolium (NBT) to NBT-diformazan according to the vendor’s protocol (R&D Systems). The extent of reduction in the appearance of NBT-formazan was used as a measure of SOD activity present in the plasma.

### 4.6. Measurement of Lactate Dehydrogenase Activity (LDH)

LDH activities in collected supernatant were determined by UV assay using a commercially available kit (Roche, Basel, Switzerland).

### 4.7. Assessment of ATPase Activity

Erythrocyte ATPase activity was measured using an ATPase assay kit (Abcam, CA, USA) as per manufacturer’s instruction.

### 4.8. Measurement of Intracellular Calcium

Intracellular Ca^2+^ was measured in incubated medium of erythrocytes treated with either vehicle (normal saline) or various concentrations of SiNPs according to a previously described technique [72]. Briefly, erythrocytes were washed with saline (0.9% NaCl) four times followed by centrifugation for 10 min at 1200× *g*. The erythrocytes were then resuspended in 1 mM Ringers solution. The final suspension consisted of 0.5% hematocrit of erythrocyte in 1 mM Ringers solution. The cells were then incubated in a flat bottom plate with the SiNPs at 37 °C for 4 h. After that, the incubated cell suspensions were collected in Eppendorf tubes and centrifuged for 5 min at 1200× *g*. The supernatant was discarded, and the cells resuspended in 5 mM Ringers solution to which 5 μL of Fura 2AM (Calbiochem; La Jolla, CA, USA) was added and incubated for 15 min at 37 °C in dark on a shaker. After that, the cell suspensions were centrifuged at 1200× *g* for 5 min. The Fura 2AM-loaded erythrocytes were resuspended in 1 mM Ringers solution and incubated for 30 min at 37 °C in dark on a shaker. Finally, Ca^2+^-dependent fluorescence intensity was then monitored with a fluorometer (model SFM 25, Kontron; Zurich, Switzerland) set at 340 nm excitation and 510 nm emission [73].

### 4.9. Assessment of Annexin V

Annexin V bound to exposed phosphatidylserine was measured in incubated medium of erythrocytes using a mouse ANXA5 ELISA kit according to the manufacturer’s instruction (Elabscience, Houston, TX, USA).

### 4.10. Measurement of Calpain Activity

The activated calpain in the cytosol of incubated erythrocytes was fluorometrically determined using a calpain activity assay kit according to the manufacturer’s instruction (Genway Biotech, San Diego, CA, USA).

### 4.11. Statistics

All statistical analyses were performed with GraphPad Prism Software version 7.05 (San Diego, CA, USA) and the data and figures were reported as mean ± SEM. Comparisons between groups were performed by one-way analysis of variance (ANOVA) followed by the Holm-Sidak multiple comparisons test. *p*-values < 0.05 were considered significant.

## Data Availability

The data that support the findings of this study are available from the corresponding author, Abderrahim Nemmar, upon reasonable request.
